# Be bold, start cold! cold formalin fixation of colorectal cancer specimens granted superior DNA and RNA quality for downstream molecular analysis

**DOI:** 10.1007/s00418-024-02326-5

**Published:** 2024-09-24

**Authors:** Ennio Nano, Alessandro Gambella, Michele Paudice, Anna Garuti, Simona Pigozzi, Luca Valle, Federica Grillo, Luca Mastracci

**Affiliations:** 1 Molecular Pathology Unit, IRCCS San Martino Policlinic Hospital of Genoa, Genoa, Italy; 2 Pathology Unit, Department of Surgical Sciences and Integrated Diagnostics (DISC), University of Genoa, Genoa, Italy; 3 IRCCS San Martino Policlinic Hospital of Genoa, Genoa, Italy; 4 Internal Medicine Clinic, IRCCS San Martino Policlinic Hospital of Genoa, Genoa, Italy

**Keywords:** Sample preservation, Molecular analysis, Quality assurance, Cold fixation

## Abstract

The use of cold formalin fixation (CFF; i.e., fixating tissue samples with 4 °C precooled formalin) recently attracted further attention owing to its putative improved ability to preserve nucleic acid compared with standard room temperature formalin (SFF). In this study, we aimed to assess the effect of four formalin-based fixation protocols (SFF, CFF, delayed formalin fixation-DFF, and cold formalin hyperfixation; CFH) on both DNA and RNA quality. We collected 97 colorectal cancer (CRC) and analyzed 23 metrics of nucleic acid quantity and quality yield using a multiplatform approach by combining spectrophotometric, fluorimetric, electrophoretic, and polymerase chain reaction (PCR) assays. Following confirmation of fixation-protocol-related different effects via clustering analysis, CFF presented best metrics compared with all protocols, specifically positive coefficients of DV1000-60000, DV2/DV1, DNA λ ratio 260/230, and *ABL* gene expression absolute copies, and negative coefficient of DV150-1000. The SFF subgroup presented a positive coefficient of DV150-1000 and negative coefficients for DV1000-60000, DV2/DV1, RNA λ ratio 260/230, RNA QuBit concentration, DV100/200, RNA electrophoresis concentration and absolute quantity, and *ABL* copies. Overall, we confirmed the superior yield performances of CFF preservation for both DNA and RNA compared with the other protocols in our series of CRC samples. Pending further validations and clarification of the specific mechanisms behind these findings, our study supports the implementation of CFF in the pathology unit routine specimen management for tumor tissue molecular profiling.

**Supplementary Information:**

The online version contains supplementary material available at 10.1007/s00418-024-02326-5.

## Introduction

This study originated from the recent keen interest in using cold formalin fixation (CFF, i.e., fixating tissue samples with 4 °C precooled formalin) to improve nucleic acid yield for downstream molecular analysis.

CFF and related tissue processing were already described in mid-1900s for horseradish-peroxidase-based immunohistochemistry stains (Straus 1968, 1980). Since then, several studies demonstrated superior immunohistochemical staining with CFF (Pollard et al. 1987; De Rosa et al. 1987; Ozzello et al. 1991; Bass et al. 2014), but recently the interest in CFF protocol revamped to solve pre-analytical issues associated with standard room temperature formalin fixation (SFF), especially in the molecular analysis domain (Bussolati et al. 2011; Do and Dobrovic 2015; Berrino et al. 2020). The adverse effects of SFF on DNA and RNA include (extensive) nucleic acid fragmentation and degradation (Chung et al. 2006; Didelot et al. 2013; Wong et al. 2014; Hedegaard et al. 2014; Guyard et al. 2017) and formaldehyde-induced crosslinks and covalent structural modifications (Feldman 1973; McGhee and von Hippel 1975, 1977; Masuda et al. 1999; Zhang et al. 2017). Together, these alterations may hamper the results of expensive and time-consuming molecular profiling of tumor samples and, more importantly, affect the related patients’ clinical and therapeutic management (Bettoni et al. 2017; Cappello et al. 2022). Indeed, with the advent and affirmation of precision medicine and targeted therapy, the need for high-quality and reliable tumor molecular characterization become pivotal, especially for those malignancies with available and effective molecular-based therapeutic protocols, such as colorectal cancer (CRC) (Karapetis et al. 2008; Roelands et al. 2023; Dang et al. 2024; Ouladan and Orouji 2024).

In this context, the rationale behind using CFF is mainly related to the effect of lower temperature on enzyme kinetics, especially the hindrance to degrading enzymes and their effect on nucleic acid (Bass et al. 2014; Lerch et al. 2020). As several studies reported increased yield of high-molecular-weight DNA (Noguchi et al. 1997; Berrino et al. 2020) and superior quality and quantity of RNA (Bussolati et al. 2011) following precooled 4 °C formalin fixation compared with SFF, more evidence is still required before considering CFF implementation in the pathology unit routine specimen management for tumor tissue molecular profiling.

On the basis of these considerations, we sought to test and compare different fixation protocols, including CFF and SFF, to determine the related nucleic acid quality and quantity yield for downstream molecular analysis. To this aim, we used several approaches, including spectrophotometric, fluorimetric, electrophoretic, and polymerase chain reaction (PCR) techniques and related metrics on a single-institution series of 97 CRC samples.

## Materials and methods

### Samples selection and stratification

In this study, we collected and evaluated an overall cohort of 97 CRC samples surgically resected at the IRCCS San Martino Polyclinic Hospital and processed at the pathology unit of the University of Genoa. All CRC samples were not subjected to neoadjuvant therapy and had a cold ischemia time of less than 30 min. Samples were anonymized by a member of our unit who was not directly involved in this study before proceeding further with the analysis. The samples were matched for patients across fixation protocols to mitigate inter-individual tumor heterogeneity and mask potential treatment effect bias. Then, they were stratified into four subgroups on the basis of the fixation protocol. In particular, 43 samples were fixed with the SFF protocol, 34 samples according to the CFF protocol, 11 samples were incubated at room temperature for 4 h with no medium (air-exposed) before proceeding further with the SFF (delayed formalin fixation; DFF), and nine according to the cold hyperfixation protocol (cold formalin hyperfixation; CFH). Details of the fixation protocols are reported in Table 1.

**Table 1:** Details of the fixation protocols tested in this study

Fixation method	Protocol
SFF—standard formalin fixation	24-h fixation in 4% neutral-buffered formalin at room temperature, followed by automatic processing
CFF—cold formalin fixation	24-h fixation in pre-cooled (4 °C) 4% neutral-buffered formalin, followed by 4-h dehydration in precooled (4 °C) 95% ethanol, and then automatic processing deprived of the first ethanol 95% step
DFF—delayed formalin fixation	4-h room temperature incubation with no medium (air-exposed) preceding 24-h fixation in 4% neutral-buffered formalin at room temperature, followed by automatic processing
CFH—cold formalin hyperfixation	72-h fixation in precooled (4 °C) 4% neutral-buffered formalin, followed by 4-h dehydration in precooled (4 °C) 95% ethanol, and then automatic processing deprived of the first ethanol 95% step

Following the fixation and processing steps, all tissue samples were paraffin-embedded, and a hematoxylin-and-eosin (HE)-stained section was evaluated to confirm overall sample adequacy. A ratio of at least 40 tumor cells per 100 overall tissue cells was required for each sample. Furthermore, several histochemical stains were performed to assess their yield depending on the protocol adopted. Specifically, Alcian blu-periodic acid Schiff (AB-PAS), periodic acid methenamine silver (PAM), reticulin, and trichrome stains were performed (Supplementary Fig. 1) and evaluated by an expert pathologist for adequacy. All histochemical stains were performed with the Ventana BenchMark Special Stains system (Ventana Medical Systems, Tucson, AZ) using ready-to-use reagents and following manufacturer’s standardized protocols.

All procedures were in accordance with the ethical standards of the human experimentation institutional review board (IRB) of the University of Genoa/IRCCS Ospedale Policlinico San Martino (IRB approval number: 101/2021) and in accordance with the World Medical Association Declaration of Helsinki of 1964 and later versions. All patients who underwent surgical resection in our institution signed written informed consent for research purposes.

### Nucleic acid purification protocol

DNA and RNA purification were performed with the Maxwell RSC DNA formalin-fixed paraffin-embedded (FFPE) and Maxwell RSC RNA FFPE kits in a Promega Maxwell RSC extractor (Promega Corporation, 2800 Woods Hollow Road, Madison, WI 53711-5399 USA). Sample duplicates were collected in centrifuge tubes and deparaffinized by adding 300 μl of mineral oil, followed by two incubations at 56 and 80 °C in heating blocks. Tissue digestion was carried out by admixing lysis buffer and proteinase K solution to each sample tube, respectively incubated at 56 °C for 30 min and 80 °C for 4 h in heating blocks. Then, 10 μl of RNase or DNase solution were added to the nucleic-acid-containing aqueous phase of each tube. The aqueous phase was then transferred from each tube to a Maxwell FFPE Cartridge to perform automated nucleic acid extraction. Purified DNA and RNA were eluted in 55 μl nuclease-free water.

### DNA and RNA quantitative and qualitative analysis

Specific metrics of DNA and RNA quality were assessed. In particular, 22 descriptors of nucleic acid preservation quality (11 for DNA and 11 for RNA) and sample overall normalized cellularity were analyzed and quantified as detailed below and summarized in Table 2.

**Table 2:** Metrics of overall sample and nucleic acid preservation quality evaluated in our study

Target	Platform	Descriptor
DNA	ND1000 spectrophotometer	λ 260/280 ratio
		λ 260/230 ratio
		Concentration
		Absolute quantification
	Qubit fluorometer	Concentration
		Absolute quantification
	Agilent Tapestation 2200	DV 150/1000
		DV 1000/60000
		DV2/DV1
		Concentration
		Absolute quantification
RNA	ND1000 spectrophotometer	λ 260/280 ratio
		λ 260/230 ratio
		Concentration
		Absolute quantification
	Qubit fluorometer	Concentration
		Absolute quantification
	Agilent Tapestation 2200 electrophoresis	DV 100/200
		DV 200/3000
		Concentration
		Absolute quantification
	Reverse-transcriptase RT-qPCR (Elitech kit)	*ABL* copies
All sample	Normalized cellularity	

First, collected samples were quantified via the ND1000 spectrophotometer calibrated for DNA and RNA analysis. Simultaneously, contamination by chaotropic agents and proteins was assessed by measuring 260/230 and 260/280 λ ratios, respectively. Samples were then analyzed with Qubit dsDNA and RNA HS Assay kits protocols in a Qubit Fluorometer 3.0 (Life Technologies Corporation; 29,851 Willow Creek Road; Eugene, Oregon, USA). Additionally, the distribution of nucleic acid fragment size of each sample was evaluated with Agilent Tapestation 2200, and nucleic acid concentration was estimated to compare results with spectrophotometric and fluorimetric analyses. DNA and RNA analyses were performed using the Genomic DNA ScreenTape kit and the RNA HS ScreenTape kit. The absolute quantification assay of DNA and RNA was performed with a 55 μl elution volume. Then, 1 μl of extracted DNA and 1 μl of extracted RNA was mixed with 5 μl of fluorochrome-containing buffer in the respective strip tube. The tube was vortexed for 2 min, then incubated in a thermal cycler for 3 min at 72 °C. After this incubation step, the tube was rapidly transferred to an ice bath and cooled for 2 min. Strip-tube was subsequently loaded into the Agilent instrument setting electrophoretic run. Samples were batched in groups of 16 and simultaneously analyzed. Internal ladders were run with samples in each electrophoretic assay to perform absolute and semi-quantification of target fragment-size ranges. Densitometric electropherograms were analyzed by integrated Tapestation Analysis Software A.02.02 (SR1; Agilent Technologies, Inc. 2017). Data evaluated for each sample regarded percentage surface ratios between fragment size ranges and total electropherogram surface. Specifically, fragments sizes analyzed for DNA included 150-1000 bp (DV150-1000) and 1000-60000 bp (DV1000-60000) ranges and their defined ratio (DV1000-60000)/(DV150/1000) indicated as DV2/DV1. RNA quality parameters included 100-200 bp (DV100-200) and 200-3000 bp (DV200-3000) ranges. Concentration and absolute quantification were collected as well.

RNA was additionally assessed through absolute gene expression quantification of the *ABL* housekeeping gene via a reverse-transcriptase RT-qPCR with Elitech kit. Every sample was analyzed in duplicate and quantified through a four-dot standard curve: standards of 10^2^, 10^3^, 10^4^, and 10^5^ *ABL* copies defined the model’s domain. Per protocol, premix, master mix, and retro-transcriptase were thawed at room temperature and admixed within 10 min. Then, 20 μl of the final mix was mixed to 10 μl of each RNA sample in a PCR plate of 96 tubes. Dilution of samples was not necessary considering a related overall mean concentration of 234.5 ng/μl, as measured by Nanodrop, and a protocol suggested range of 80 ng/μl to 400 ng/μl to optimize target amplification. Subsequently, samples were loaded in the thermocycler, setting the thermal profile of the run and gene quantification threshold as indicated by the technical bulletin. Finally, normalized cellularity (i.e., normalization of cellularity over sample dimensions) was also evaluated to prevent potential bias during protocol comparison (Supplementary Table 1).

### Statistical analysis

Statistical analysis was performed using R Software (version 4.2.2; The R Foundation for Statistical Computing, Vienna, Austria) and RStudio (version 2022.12.0 + 353; RStudio, Boston (MA), USA). The rcmdr (version 2.7-1) and mdatools packages were used to evaluate and compare nucleic acid metrics across fixation protocols. Specific tests and related use are specified and detailed in the related Manuscript session, as appropriate.

## Results

### Descriptors and related rationale for nucleic acid quality and quantity assurance

To make reliable inferences about the impact of fixation protocols on nucleic acid quality and quantity yield, we decided to perform a broad yet specific analysis using several approaches, including spectrophotometric, fluorimetric, electrophoretic, and PCR techniques.

Specifically, we used ND1000 spectrophotometer to quantify DNA and RNA (both concentration and absolute quantification) and contamination of protein (absorbance λ ratios at 260/280) and chaotropic (absorbance λ ratios at 260/230) interferents.

Subsequently, we additionally quantify DNA and RNA (both concentration and absolute quantification) with the Qubit 3.0 fluorometer, implementing specificity in assessing fragment sizes eligible for successful amplification assays.

Furthermore, considering its relevance for downstream next-generation sequencing (NGS) analysis, quantification of DNA and RNA integrity was evaluated with Tapestation Agilent 2200 electrophoresis assay, referring to specifics of Illumina Target Panel protocol implemented in a routine laboratory setting. DNA size ranges collected included the DV1000-60000 (descriptor of DNA integrity) and DV200-3000 (descriptor of DNA degradation). Size ranges collected for RNA included the DV150-1000 (descriptor of RNA integrity) and DV100-200 (descriptor of RNA fragmentation).

RNA eligibility for amplification assays was further assessed by the highly specific *ABL* house-keeping gene expression assay as a measure of RNA quantity, integrity, and adduct damage by formalin fixation.

Ultimately, we evaluated normalized cellularity (normalization of cellularity over sample dimensions) as a broad metric of tissue specimen quality to account for potential bias during the comparison of fixation protocols. We also evaluated histological structure preservation via histochemical stains, including AB-PAS, PAM, reticulin, and trichrome stains. All of them resulted adequate by an expert pathologist assessment (Supplementary Fig. 1).

### Principal component analysis (PCA) showed fixation protocol-dependent samples clustering

**Fig. 1:**
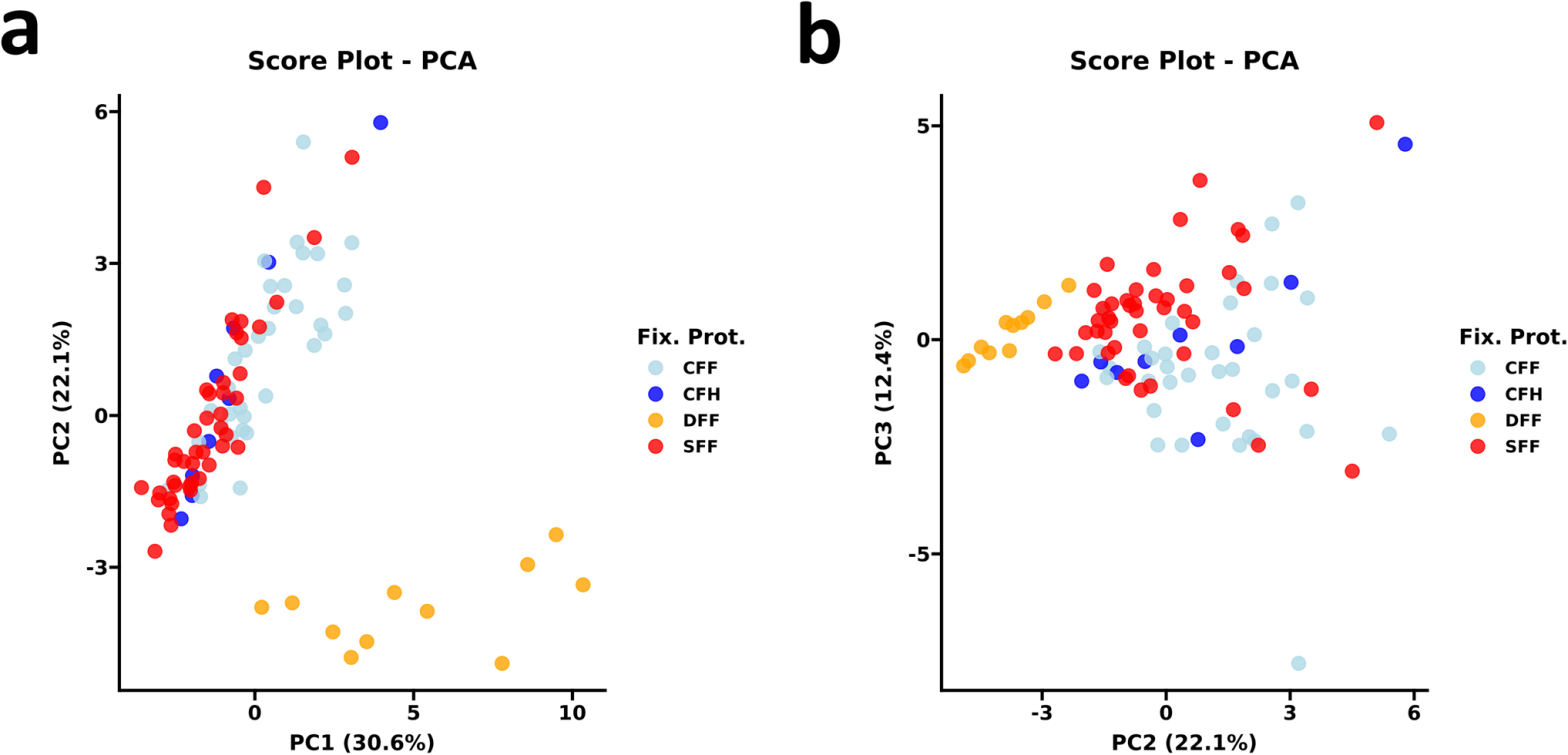
Principal component analysis (PCA) pattern recognition based on fixation protocols. PCA account for 65.1% of dataset variance in the first three dimensions. *CFF* cold formalin fixation, *CFH* cold formalin hyperfixation, *DFH* delayed formalin fixation, *FP* fixation protocols, *PC1* principal component 1, *PC2* principal component 2, *PC3* principal component 3, *SFF* standard formalin fixation

Firstly, we inspected the relationship between all the analyzed descriptors via a correlation matrix and related Pearson correlation analysis. Considering the non-negligible correlation observed between variables (Supplementary Fig. 2) and to investigate data patterns further, we decided to perform a principal component analysis (PCA. Inspecting the scree plot (Supplementary Fig. 3), we identified an elbow at the fourth component, suggesting decreased dimensional complexity of the PCA model to the first four principal components (PCs). Considering that 75% of dataset total variance was achieved with the first four PCs, the score plots revealed significant information on fixation protocols on the first 3 PCs, which accounted for 65.1% of dataset total variance. In particular, the first PC (30.6% of dataset variance) showed DFF subgroup partial clustering that was completely definite on the 2° PC. On the 3° PC, SFF and CFF clustered independently, while CFH clustered near the intersecting area of previous groups with a tendency to lay in the CFF proximity. This pattern suggested that CFH harbored features intermediate between CFF and SFF but closer to CFF, as expected (Fig. 1).

### Partial-least squares discriminant-analysis (pls-DA) confirmed samples clustering and showed stronger discriminant power than PCA

**Fig. 2:**
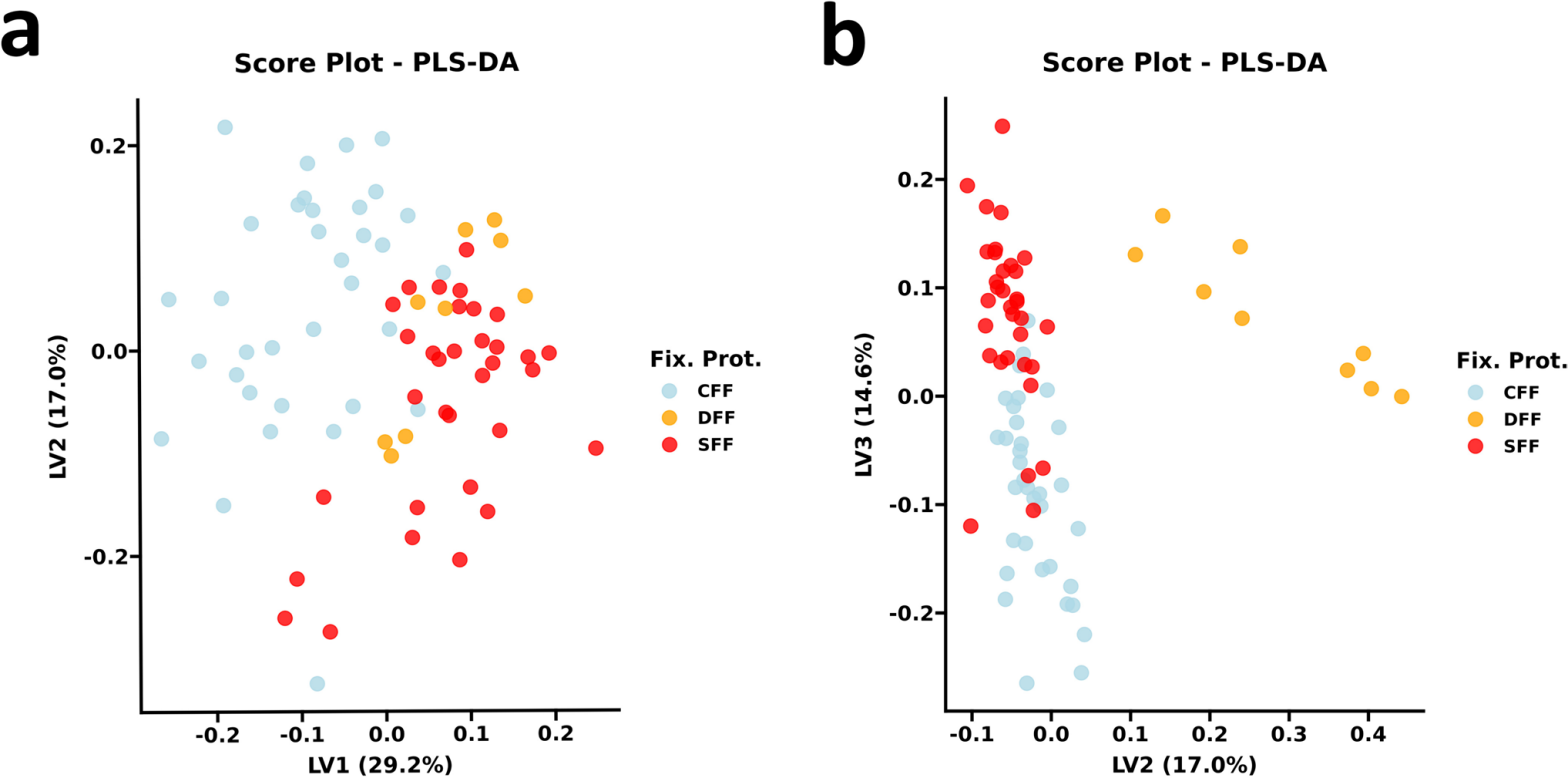
Pattern recognition based on score plot from predictors matrix of pls-DA. Pls-DA model account for 60.8% of dataset variance in the first three dimensions. Of note, pls-DA model shows stronger discriminant power than PCA for CFF and SFF classes, from the perspective of the 2° and 3° dimensions. *CFF* cold formalin fixation, *DFF* delayed formalin fixation, *Fix. Prot.* fixation protocols, *LV1* latent-variable 1, *LV2* latent-variable 2, *LV3* latent-variable 3

To further confirm protocol discrimination and to quantitatively assess each variable weight on the definition of each fixation subgroup, we built an in-fitting and in-cross-validation classification supervised model based on a partial-least squares discriminant-analysis (pls-DA). We computed the Pls-DA model on the basis of the clustering pattern modeled by PCA, and decided to merge CFH and CFF samples owing to their partial overlap. The dataset was subdivided into a calibration set (76 samples) and a validation set (21 samples) by randomly assigning samples to the fitting and prediction sets with a ratio of 8:2 assignments for every supervised class. The selection of model-defining latent-variable (LV) number was based on a root-mean-square-error (RMSE) graph, plotting RMSE against model defining LV number and using the lowest RMSE to define the criteria of LV number selection. The first five LVs, accounting for 75,12% of explained dataset variance, described the pls-DA model best cross-validation performance, computed in a “leave-one-out” setting. On the basis of five LVs, the CFF class showed a sensitivity of 85,3%, a specificity of 97,6%, and an overall accuracy of 92,1%. Top classification performance was evidenced for the DFF class with a specificity, sensitivity, and accuracy of 100%. SFF class showed an 87,9% sensitivity, a specificity of 88,4%, and an accuracy of 88,2%. The validated pls-DA model, described by the first two LVs, showed the best predictive performance. The CFF class harbored a sensitivity of 88,9%, a specificity of 91,7%, and an accuracy of 90,5%, while the SFF class showed a sensitivity of 90%, a specificity of 90,9% and an accuracy of 90,5%. Only the first LV was sufficient to reach maximum classification performances for the DFF class. For each supervised class, model coefficients were computed, selecting the LVs with the best predictive performance. In particular, the first two LVs for the CFF and SFF subgroups, and the first LV for the DFF subgroup (Fig. 2).

### Inference tests identified descriptors driving fixation protocol clustering

**Fig. 3:**
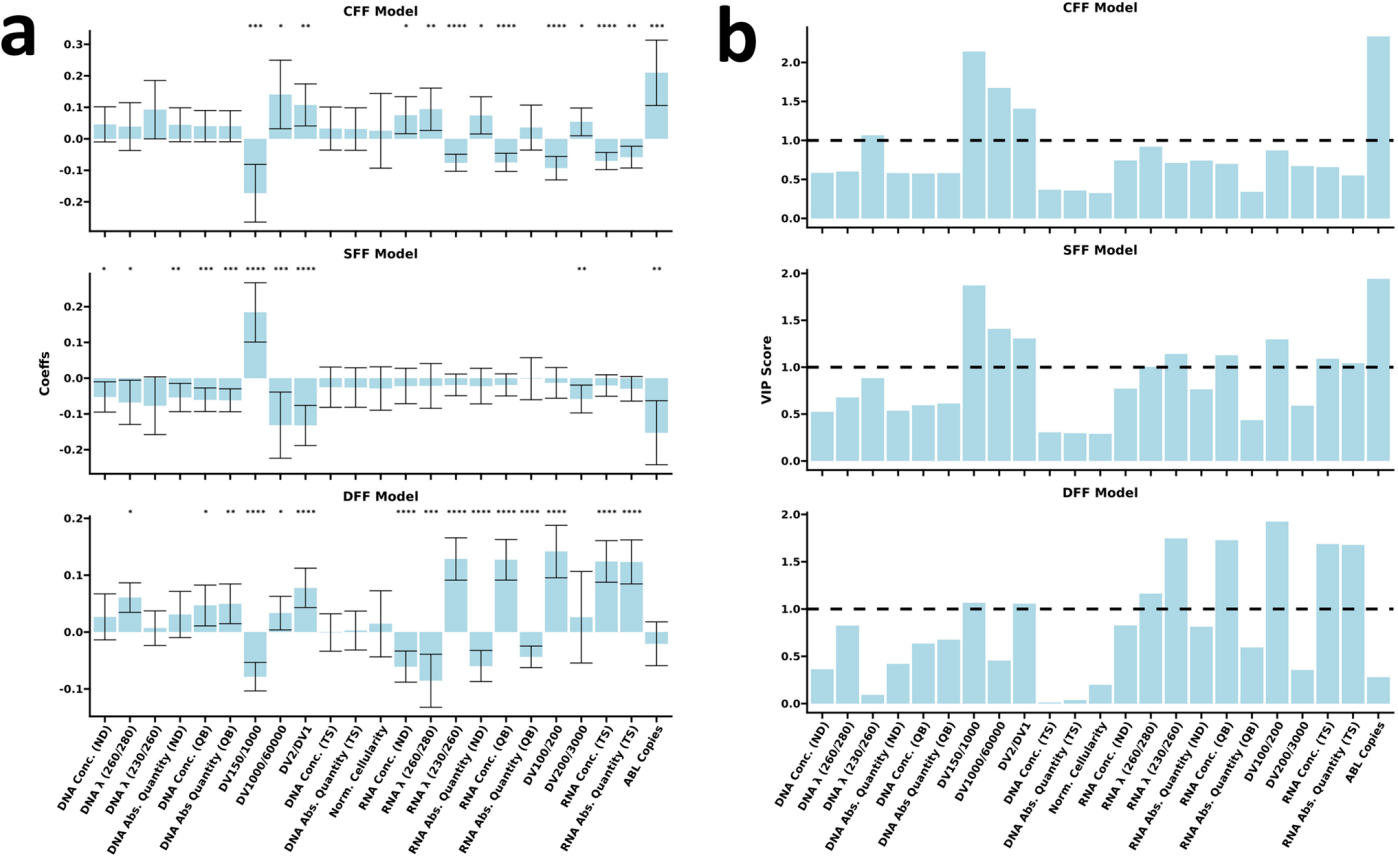
Regression coefficients with respective 95% C.I. and VIP scores show to be partially concordant across all classes in terms of significance *p < 0.05; **p < 0.01; ***p < 0.001; ****p < 0.0001; VIP scores threshold of significance set at 1 (dotted line). *DNA.Conc. (ND)* DNA concentration (ND1000 spectrophotometer), *DNA.λ (260/280)* DNA λ 260/280 ratio (ND1000 spectrophotometer), *DNA.λ (260/230)* DNA λ 260/230 ratio (ND1000 spectrophotometer), *DNA.Abs.Quantity (ND)* DNA absolute quantification (ND1000 spectrophotometer), *DNA.Conc. (QB)* DNA concentration (Qubit fluorometer), *DNA.Abs.Quantity (QB)* DNA absolute quantification (Qubit fluorometer), *DNA.Conc. (TS)* DNA concentration (Agilent Tapestation 2200), *DNA.Abs.Quantity (TS)* DNA absolute quantification (Agilent Tapestation 2200), *Norm.Cellularity* Normalized cellularity, *RNA.Conc. (ND)* RNA concentration (ND1000 spectrophotometer), *RNA.λ (260/280)* RNA λ 260/280 ratio (ND1000 spectrophotometer), *RNA.λ (260/230)* RNA λ 260/230 ratio (ND1000 spectrophotometer), *RNA.Abs.Quantity (ND)* RNA absolute quantification (ND1000 spectrophotometer), *RNA.Conc. (QB)* RNA concentration (Qubit fluorometer), *RNA.Abs.Quantity (QB)* RNA absolute quantification (Qubit fluorometer), *RNA.Conc. (TS)* RNA concentration (Agilent Tapestation 2200), *RNA.Abs.Quantity (TS)* DNA absolute quantification (Agilent Tapestation 2200), *ABL.Copies ABL* gene copies (reverse-transcriptase RT-qPCR; Elitech kit)

Reassured that nucleic acid descriptors clustered based on fixation protocols, we decided to evaluate the weights of each descriptor and the relative significance. For this purpose, we used two inference tests. The jack-knife test estimated the mean of every variable coefficient and its confidence interval (CI) from the respective distribution computed through a “leave-one-out” cross-validation. Significant variables were selected to define each model class with variables important for projection (VIP) scores computation in a “leave-one-out” cross-validation setting, specifying a threshold of 1. Both jack-knife CI and VIP scores substantially showed concordant results and were compared for the reproducibility of model features. Detailed outcomes of the jack-knife test and VIP score are reported in Fig. 3.

Five significant variables—DV150-1000, DV1000-60000, DV2/DV1, DNA λ ratio 260/230, and ABL absolute copies—describe the CFF class model. All variables show positive coefficients, with the coherent exception of DV150-1000. Seven variables are identified as significant in the SFF class: DV150-1000 with a positive coefficient and six others with negative trends, namely DV1000-60000, DV2/DV1, RNA λ ratio 260/230, RNA qubit concentration, DV100/200, RNA electrophoresis concentration and absolute quantity, and ABL copies. Significant variables with positive coefficients for the DFF class were DV2/DV1, RNA λ ratio 260/230, RNA qubit concentration, RNA electrophoresis concentration, and absolute quantity, and DV100-200. DV150-1000 and RNA λ ratio 260/280 also negatively weighed the DFF class. Box plots showing the results of univariate analysis specifically comparing the impact of fixative protocols on each metric of DNA and RNA quality are reported in Supplementary Fig. 4 and Supplementary Fig. 5, respectively.

## Discussion

In this study, we tested different formalin-based fixative protocols and demonstrated that fixation at lower temperatures yielded DNA and RNA with improved quality and quantity.

We used 97 samples of CRC to test four different fixation conditions (SFF-standard formalin fixation, CFF-cold formalin fixation, DFF-delayed formalin fixation, and CFH-cold formalin hyperfixation) and thoroughly characterized nucleic acid yield via spectrophotometric, fluorimetric, electrophoretic, and PCR techniques and related metrics. As our cohort of CRC samples was not screened for specific morphological and genomic profiles, we opted for chemometric approaches, namely PCA and pls-DA, to discriminate the effects of fixation protocols. First, we demonstrated that DFF, CFF, and SFF samples presented significantly different quality metrics, as expected. Of note, the CFH subgroup did not cluster independently but was instead localized in an overlapping area between CFF and SFF clusters, with a tendency to be closer to the CFF subgroup. These results suggest that prolonged fixation nullified the effects of the low-temperature environment, but the lack of CFH independent clustering may also have been related to the small sample size of this subgroup.

Granular analysis of the metrics that defined fixation protocols clustering provided interesting data on RNA yield. In our study, RNA 260/280 λ ratio positively weighed on the CFF model, while *ABL* copies and RNA 260/280 λ ratio were inversely correlated with SFF. These results suggest that crosslinking kinetics largely relies on fixative temperature and support the hypothesis that, compared with SFF, CFF could (1) reduce the formalin-mediated crosslinking within and between RNA strands and between RNA strands and proteins, and (2) hinder formalin-mediated RNA distortion implying reduced retro-transcriptase inhibition and protein contamination. Furthermore, our results suggest that RNA fragmentation did not represent a relevant mechanism of formalin-mediated degradation. In our series, DV100-200 and DV200-3000 marginally influence the CFF and SFF models according to VIP score and C.I. diagnostics. Our data are in line with literature. Masuda and colleagues described formalin-mediated intra- and inter-strand crosslinking, where formalin reacted with ammine moieties of singular or adjacent nucleobases, resulting in mono-methylol ammine adducts and methylene bridges, respectively (Masuda et al. 1999). Kennedy-Darling and colleagues described several formalin-dependent crosslinking mechanisms, the most relevant involving the nucleophilic link of Lysine-rich protein ammino-groups to the electrophilic carbon of formalin (with the formation of an intermediate Schiff base), followed by the nucleophilic link of a deoxyguanosine ammine moiety to the Schiff-base. The result is an aminal bridge formation between the protein and the nucleobase (Kennedy-Darling and Smith 2014). Authors inferred that formalin crosslinking rate was positively associated with temperature (Kennedy-Darling and Smith 2014). Similarly, Chafin and colleagues observed low formalin-mediated crosslinking at cold temperatures and indicated temperature-dependent reduction of reaction kinesis as the putative explanation (Chafin et al. 2013). Ultimately, our findings support and further refine the literature evidence that (1) formalin-mediated crosslinking is the most relevant mechanism of RNA modifications, and (2) this mechanism is temperature-dependent and can be mitigated by CFF.

Focusing on the DNA analysis, in our cohort, positive coefficient predictors of CFF included DV1000-60000 and DV2/DV1, whereas DV150-1000 was a negative coefficient predictor. Furthermore, the DNA λ 260/280 ratio was barely significant on SFF, thus suggesting that differences between CFF and SFF are unrelated to DNA purity. Overall, this data supports the superior metrics of CFF compared to SFF, as previously reported in the literature (Berrino et al. 2020). Proposed mechanisms involved inverse trends of DV150-1000 and DV1000-60000 predictors comparing CFF with SFF models (Berrino et al. 2020), as confirmed in our study.

Regarding the sample type, we decided to analyze CRC specimens owing to (1) the clinical relevance of CRC reliable molecular profiling for patients therapeutic management (Karapetis et al. 2008; Roelands et al. 2023; Dang et al. 2024; Ouladan and Orouji 2024), (2) the well-known and peculiar impact of formalin fixation on nucleic acid fragmentation for this cancer type (Lamy et al. 2011; Guyard et al. 2017), and (3) the expertise of our group with the preanalytical conditions and morpho-molecular profiles of CRC (Bragoni et al. 2017; Gambella et al. 2017, 2022; Remo et al. 2019; Mastracci et al. 2019; Pitto et al. 2020; Carlin et al. 2023; Grillo et al. 2023a, 2023b, 2024b, 2024a). Still, the focus on CRC-only samples and the relatively small and monocentric series limited the impact of our findings. It is worth mentioning that there are other metrics available in literature that can be used to assess nucleic acid quality, such as the fraction of amplified DNA following qPCR reactions for NGS libraries preparation (Bettoni et al. 2017). Nevertheless, we believe that our study enriches the evidence available in the literature and kindles further evaluation on larger multicentric cohorts, especially for RNA quality assessment.

In conclusion, we demonstrated that CFF-cold formalin fixation improved both DNA and RNA preservation in our cohort of CRC samples. Our granular and cross-platform analysis requires further validation but undoubtedly indicates the superior yield of CFF compared with SFF. As we elucidate the mechanisms behind these findings, our study supports the implementation of CFF in the pathology unit routine specimen management for tumor tissue molecular profiling.

## Supplementary Information

Below is the link to the electronic supplementary material.

Supplementary file1 (DOCX 2471 KB)

## Data Availability

The dataset generated for this study is available from the authors upon reasonable request.
