# Supplementary material for: Be bold, start cold! cold formalin fixation of colorectal cancer specimens granted superior DNA and RNA quality for downstream molecular analysis
Source: Histochem Cell Biol. 2024 Sep 24;162(6):541–50. doi: 10.1007/s00418-024-02326-5 (PMC11455702; doi:10.1007/s00418-024-02326-5)
Supplement: Supplementary file 1 — Supplementary file1 (DOCX 2471 KB) [file 418_2024_2326_MOESM1_ESM.docx]

Be bold, start cold! Cold formalin fixation of colorectal cancer specimens granted superior DNA and RNA quality for downstream molecular analysis.

Ennio Nano, Alessandro Gambella, Michele Paudice, Anna Garuti, Simona Pigozzi, Luca Valle, Federica Grillo, Luca Mastracci

**Supplementary Materials**

**Index**

| **Item** | **Page** |
| --- | --- |
| **Supplementary Figure 1. Histochemical stains are adequate with different fixation protocols.** | **2** |
| **Supplementary Figure 2. Heatmap plot showing correlation across acid nucleic metrics.** | **3** |
| **Supplementary Figure 3. Scree plot showing dimensional complexity and dataset variance of the first four principal components.** | **4** |
| **Supplementary Figure 4. Box plots showing the results of univariate analysis specifically comparing the impact of fixative protocols on each metric of DNA quality.** | **5** |
| **Supplementary Figure 5. Box plots showing the results of univariate analysis specifically comparing the impact of fixative protocols on each metric of RNA quality.** | **6** |
| **Supplementary Table 1. Results of the normalized cellularity analysis across samples and protocols.** | **7-9** |


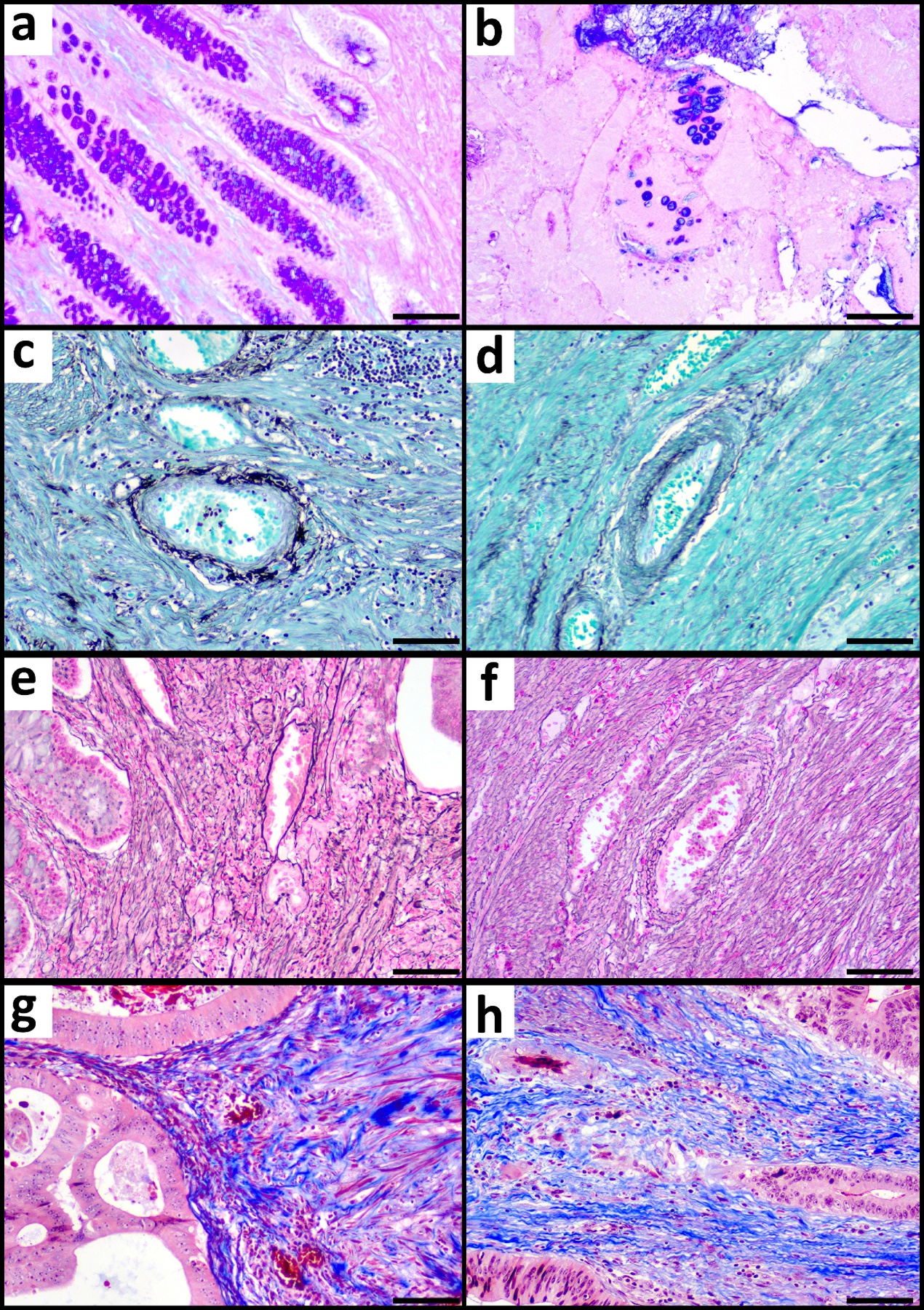


**Supplementary Figure 1. Histochemical stains are adequate with different fixation protocols.**

Alcian blu-periodic acid Schiff (a-b), Periodic acid methenamine silver (c-d), reticulin (e-f), and trichrome (g-h) stains were adequate with both the standard (a, c, e, g) and the cold (b, d, f, h) formalin fixation protocols. Scale bars: 100 µm (magnification x40).


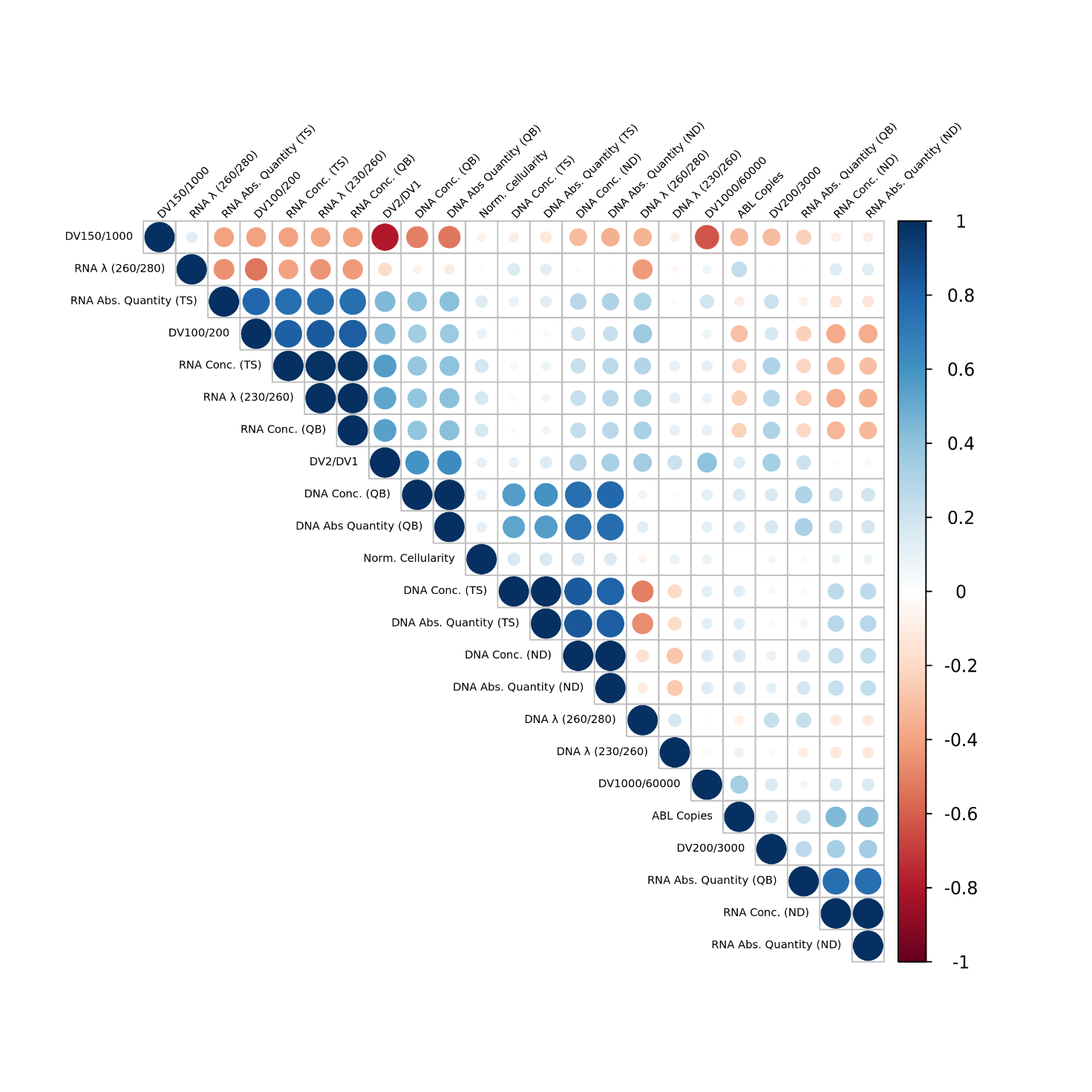


**Supplementary Figure 2. Heatmap plot showing correlation across acid nucleic metrics**

DNA.Conc. (ND): DNA concentration (ND1000 Spectrophotometer); DNA.λ (260/280): DNA λ 260/280 ratio (ND1000 Spectrophotometer); DNA.λ (260/230): DNA λ 260/230 ratio (ND1000 Spectrophotometer); DNA.Abs.Quantity (ND): DNA absolute quantification (ND1000 Spectrophotometer); DNA.Conc. (QB): DNA concentration (Qubit Fluorometer); DNA.Abs.Quantity (QB): DNA absolute quantification (Qubit Fluorometer); DNA.Conc. (TS): DNA concentration (Agilent Tapestation 2200); DNA.Abs.Quantity (TS): DNA absolute quantification (Agilent Tapestation 2200); Norm.Cellularity: Normalized cellularity; RNA.Conc. (ND): RNA concentration (ND1000 Spectrophotometer); RNA.λ (260/280): RNA λ 260/280 ratio (ND1000 Spectrophotometer); RNA.λ (260/230): RNA λ 260/230 ratio (ND1000 Spectrophotometer); RNA.Abs.Quantity (ND): RNA absolute quantification (ND1000 Spectrophotometer); RNA.Conc. (QB): RNA concentration (Qubit Fluorometer); RNA.Abs.Quantity (QB): RNA absolute quantification (Qubit Fluorometer); RNA.Conc. (TS): RNA concentration (Agilent Tapestation 2200); RNA.Abs.Quantity (TS): DNA absolute quantification (Agilent Tapestation 2200); ABL.Copies: *ABL* gene copies (reverse-transcriptase RT-qPCR; Elitech kit).


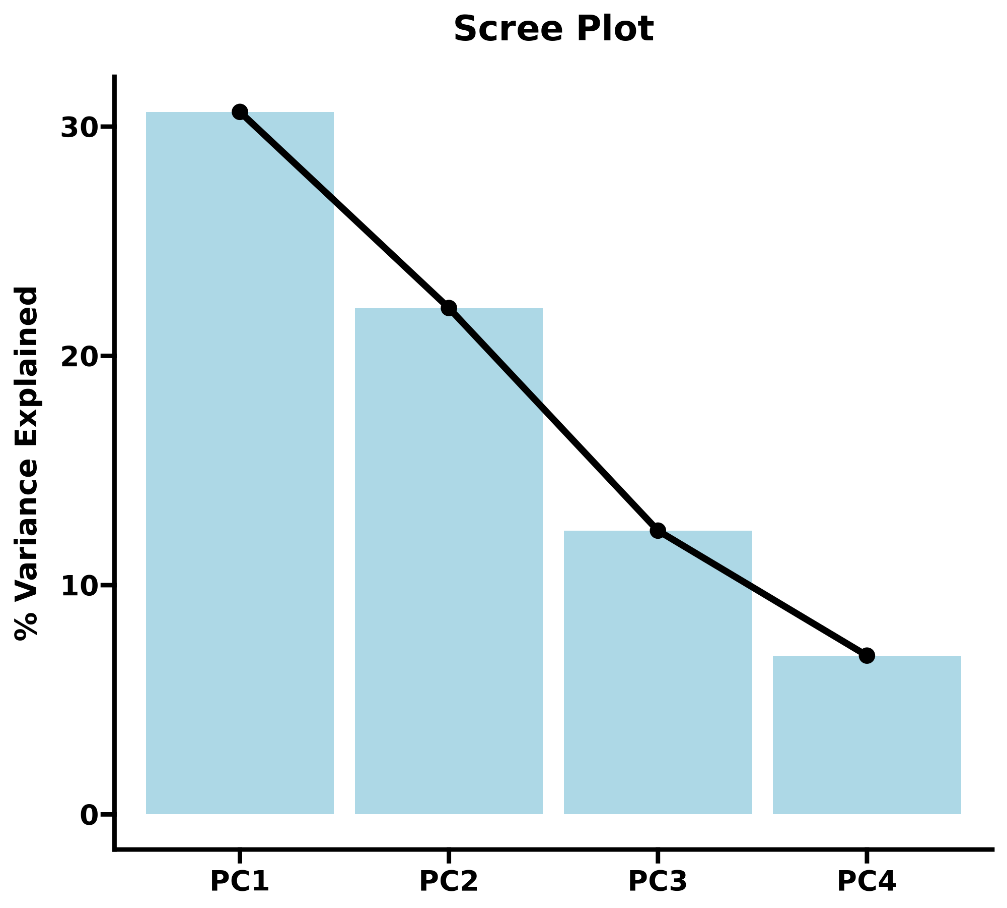


**Supplementary Figure 3. Scree plot showing dimensional complexity and dataset variance of the first four principal components**

PC1: principal component 1; PC2: principal component 2; PC3: principal component 3; PC4: principal component 4.


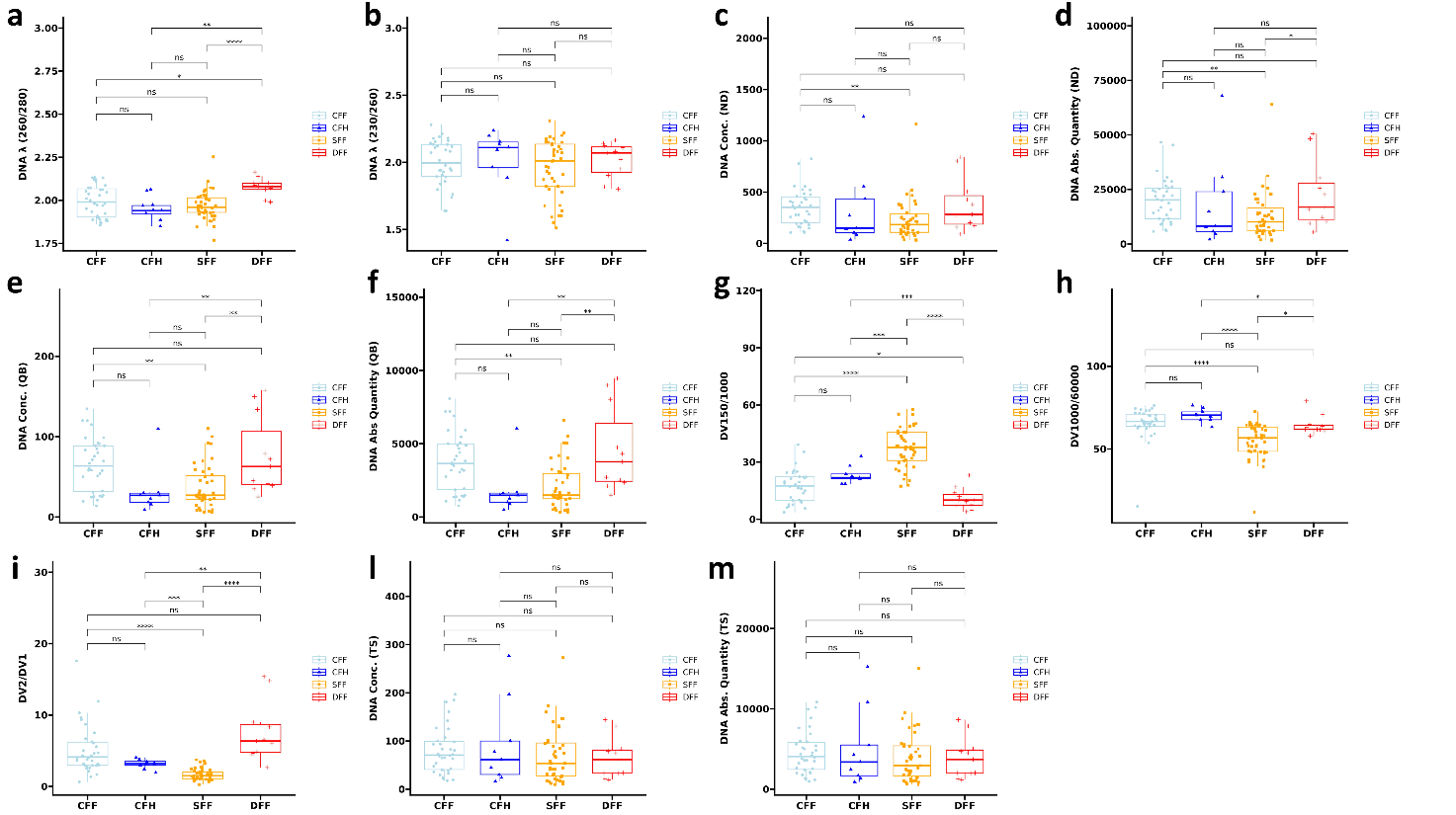


**Supplementary Figure 4. Box plots showing the results of univariate analysis specifically comparing the impact of fixative protocols on each metric of DNA quality.**

Class comparisons were carried out by means of a non-parametric test (Wilcoxon signed-rank test) due to the skewness of variable distributions and class imbalances. In each class pair tested, p-values were adjusted for multiple comparisons accordingly to Benjamini-Hochberg correction.

a) λ 260/280 ratio via ND1000 Spectrophotometer; b) λ 260/230 ratio via ND1000 Spectrophotometer; c) Concentration via ND1000 Spectrophotometer; d) Absolute quantification via ND1000 Spectrophotometer; e) Concentration via Qubit Fluorometer; f) Absolute quantification via Qubit Fluorometer; g) DV 150/1000 via Agilent Tapestation 2200; h) DV 1000/60000 via Agilent Tapestation 2200; i) DV2/DV1 via Agilent Tapestation 2200; l) Concentration via Agilent Tapestation 2200; m) Absolute quantification via Agilent Tapestation 2200. *: p<0.05; **: p<0.01; ***: p<0.001; ****: p<0.0001;

**
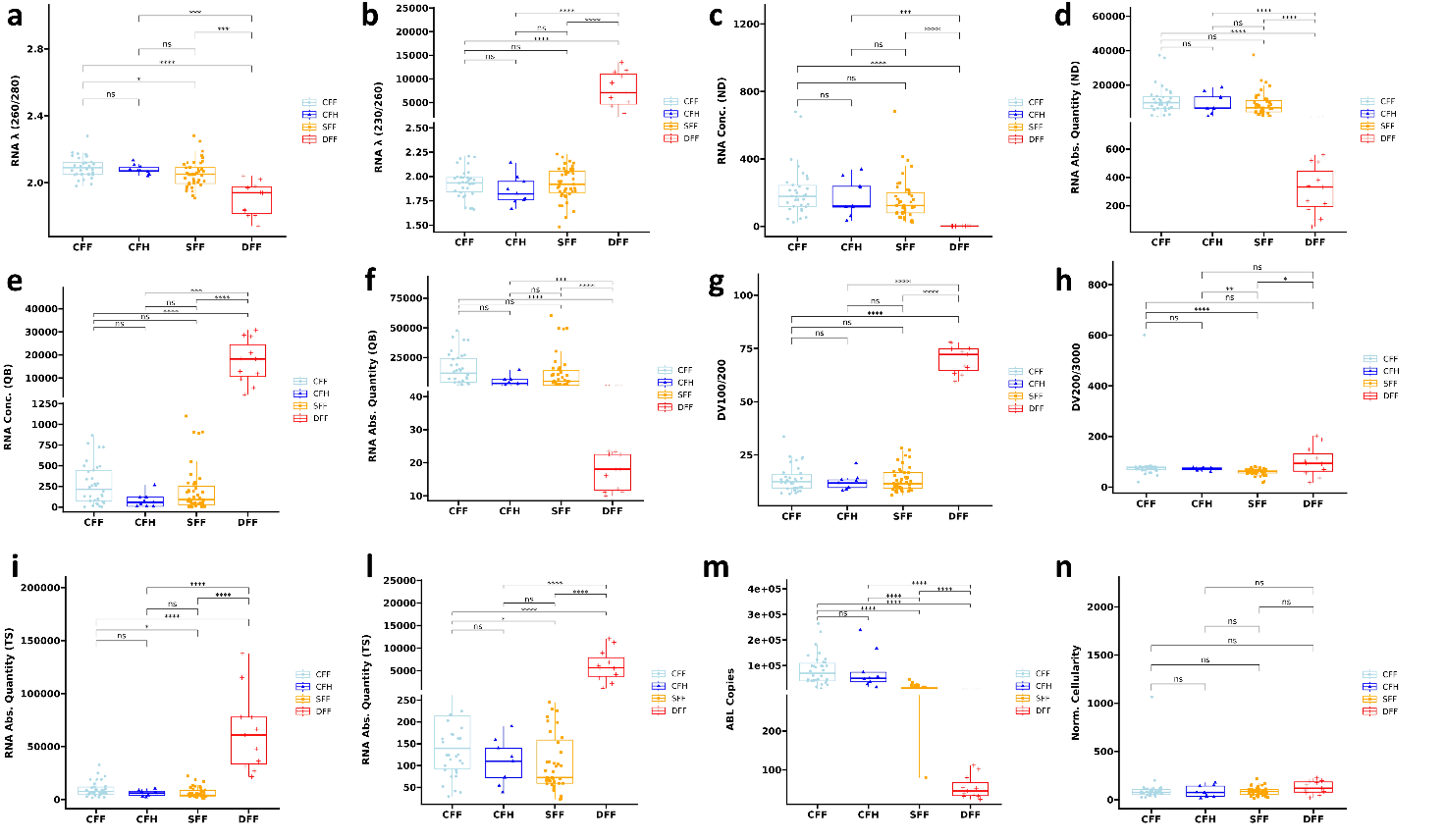
**

**Supplementary Figure 5. Box plots showing the results of univariate analysis specifically comparing the impact of fixative protocols on each metric of RNA quality.**

Class comparisons were carried out by means of a non-parametric test (Wilcoxon signed-rank test) due to the skewness of variable distributions and class imbalances. In each class pair tested, p-values were adjusted for multiple comparisons accordingly to Benjamini-Hochberg correction.

a) λ 260/280 ratio via ND1000 Spectrophotometer; b) λ 260/230 ratio via ND1000 Spectrophotometer; c) Concentration via ND1000 Spectrophotometer; d) Absolute quantification via ND1000 Spectrophotometer; e) Concentration via Qubit Fluorometer; f) Absolute quantification via Qubit Fluorometer; g) DV 100/200 via Agilent Tapestation 2200; h) DV 200/300 via Agilent Tapestation 2200; i) Concentration via Agilent Tapestation 2200; l) Absolute quantification via Agilent Tapestation 2200; m) *ABL* Absolute quantification via RT-qPCR; n) Normalized cellularity. *: p<0.05; **: p<0.01; ***: p<0.001; ****: p<0.0001;

**Supplementary Table 1. Results of the normalized cellularity analysis across samples and protocols**

| **Samples** | **Normalized Cellularity** |
| --- | --- |
| ***Cold Formalin Fixation - CFF*** | |
| CFF_1 | 72 |
| CFF_2 | 45 |
| CFF_3 | 96.85 |
| CFF_4 | 126 |
| CFF_5 | 24 |
| CFF_6 | 165.33 |
| CFF_7 | 87.5 |
| CFF_8 | 68.25 |
| CFF_9 | 84 |
| CFF_10 | 111.15 |
| CFF_11 | 107.25 |
| CFF_12 | 77 |
| CFF_13 | 34.125 |
| CFF_14 | 52.5 |
| CFF_15 | 60.2 |
| CFF_16 | 200 |
| CFF_17 | 88 |
| CFF_18 | 94.5 |
| CFF_19 | 117.6 |
| CFF_20 | 81.9 |
| CFF_21 | 126 |
| CFF_22 | 72 |
| CFF_23 | 117.6 |
| CFF_24 | 71.4 |
| CFF_25 | 81 |
| CFF_26 | 69 |
| CFF_27 | 56 |
| CFF_28 | 73.5 |
| CFF_29 | 112 |
| CFF_30 | 50.4 |
| CFF_31 | 25.2 |
| CFF_32 | 72 |
| CFF_33 | 42 |
| CFF_34 | 56 |
| **Median** | **75.25** |
| ***Cold Formalin Hyperfixation - CFH*** | |
| CFH_1 | 15 |
| CFH_2 | 150.5 |
| CFH_3 | 45 |
| CFH_4 | 74.25 |
| CFH_5 | 178.5 |
| CFH_6 | 33.15 |
| CFH_7 | 77 |
| CFH_8 | 29.7 |
| CFH_9 | 140.4 |
| **Median** | **74.25** |
| ***Delayed Formalin Fixation - DFF*** | |
| DFF_1 | 85.1 |
| DFF_2 | 196.7 |
| DFF_3 | 118 |
| DFF_4 | 70.7 |
| DFF_5 | 100.5 |
| DFF_6 | 175.4 |
| DFF_7 | 20.6 |
| DFF_8 | 225.3 |
| DFF_9 | 191.4 |
| DFF_10 | 152.8 |
| DFF_11 | 45.5 |
| **Median** | **118** |
| ***Standard Room Temperature Formalin Fixation - SFF*** | |
| SFF_1 | 41.25 |
| SFF_2 | 102 |
| SFF_3 | 156 |
| SFF_4 | 88.2 |
| SFF_5 | 79.8 |
| SFF_6 | 15 |
| SFF_7 | 76.5 |
| SFF_8 | 69 |
| SFF_9 | 138 |
| SFF_10 | 36 |
| SFF_11 | 147 |
| SFF_12 | 60 |
| SFF_13 | 60.75 |
| SFF_14 | 105 |
| SFF_15 | 85.8 |
| SFF_16 | 21 |
| SFF_17 | 216 |
| SFF_18 | 94.5 |
| SFF_19 | 94.5 |
| SFF_20 | 84 |
| SFF_21 | 88.2 |
| SFF_22 | 29.25 |
| SFF_23 | 49.5 |
| SFF_24 | 39 |
| SFF_25 | 92 |
| SFF_26 | 60.5 |
| SFF_27 | 52.5 |
| SFF_28 | 168 |
| SFF_29 | 151.2 |
| SFF_30 | 67.2 |
| SFF_31 | 105 |
| SFF_32 | 105 |
| SFF_33 | 56 |
| SFF_34 | 119 |
| SFF_35 | 81 |
| SFF_36 | 93.1 |
| SFF_37 | 84 |
| SFF_38 | 86.4 |
| SFF_39 | 45 |
| SFF_40 | 28 |
| SFF_41 | 112 |
| SFF_42 | 36 |
| SFF_43 | 80 |
| **Median** | **84** |
